# Differential Biological and Molecular Profiling of Mesenchymal Progenitor Cells in Cartilage from Osteoarthritis and Rheumatoid Arthritis: An In Vitro Study

**DOI:** 10.3390/ijms27125252

**Published:** 2026-06-10

**Authors:** Akshay Bairapura Manjappa, Narendra Nitilapura, Siddharth Shetty, Shama Rao, Santhosh Babu, Jayaprakasha Shetty, Reshma Shetty, Mohana Kumar Basavarajappa

**Affiliations:** 1 Nitte University Centre for Stem Cell Research and Regenerative Medicine, K. S. Hegde Medical Academy, Nitte (Deemed to be University), Deralakatte, Mangaluru 575018, India; akshay.bm@nitte.edu.in (A.B.M.);; 2 Department of Anatomy, K. S. Hegde Medical Academy, Nitte (Deemed to be University), Deralakatte, Mangaluru 575018, India; 3 Department of Orthopaedics, K. S. Hegde Medical Academy, Nitte (Deemed to be University), Deralakatte, Mangaluru 575018, India; 4 Central Research Laboratory, K. S. Hegde Medical Academy, Nitte (Deemed to be University), Deralakatte, Mangaluru 575018, India

**Keywords:** osteoarthritis, rheumatoid arthritis, mesenchymal progenitor cells, articular cartilage, cartilage regeneration, gene expression, cell proliferation, cell differentiation, cellular senescence

## Abstract

Mesenchymal progenitor cells (MPCs) play a significant role in articular cartilage homeostasis and regeneration. Yet, the functional dynamics and molecular characteristics of MPCs may differ significantly across various pathological conditions. Hence, this study comprehensively investigates the biological and molecular characteristics of MPCs isolated from articular cartilage of patients with osteoarthritis (OA) and rheumatoid arthritis (RA), aiming to uncover disease-specific differences that could offer insights into targeted regenerative therapies. Using flow cytometry, gene expression analysis, and in vitro differentiation assays, we assessed the phenotype, growth potential, senescence, cytogenetic instability, and chondrogenic potential to delineate molecular pathways uniquely active in each disease context. Phenotypically, both OA and RA-MPCs retained markers of mesenchymal stem cells (MSCs), but OA-derived MPCs exhibited higher fold expression of progenitor markers (*OCT-4*, *NANOG*, *SOX-2*, and *SSEA-4*), suggesting a more activated state. Functionally, OA-MPCs demonstrated increased growth kinetics (higher proliferation rate and decreased population doubling time) with a significant shift towards adipogenic lineages (increased fold expression of *LPL*, *AP2*, and *PPAR-γ*). However, there were no differences in the osteogenic and chondrogenic potential. Gene expression analysis revealed upregulation of genes involved in extracellular matrix production and cartilage development (*COL2-α1*, *ACAN, FGFR3*, *TGF-β3*, *ANXA6*, *CNTN1*, *MATN1*, *TGF-β1*, *VIM*, and *SOX9*) in 3D cultures compared with 2D or monolayer cultures. Collectively, these findings demonstrate that, while multipotent MPCs are present in both OA and RA articular cartilage, they can exhibit fundamentally altered biological behaviors and molecular signatures reflective of the local disease microenvironment. Understanding these differences is critical for optimizing cell-based therapeutic strategies tailored to each condition and may facilitate the development of novel interventions targeting endogenous progenitor cells for cartilage repair.

## 1. Introduction

Osteoarthritis (OA) and rheumatoid arthritis (RA) are known chronic inflammatory degenerative diseases agonizing divergent age cohorts globally, impacting one or more synovial joints and associated connective tissues. OA was once thought to be linked solely to ageing or trauma; however, recent research indicates the influence of lifestyle-associated variables, non-modifiable genetics, and other idiopathic factors [1,2,3,4,5,6]. RA is primarily an autoimmune disorder characterized by altered innate and adaptive immunity, leading to recurrent joint swelling and tenderness. Its progression triggers a cascade of events encompassing the infiltration of lymphocytes, upregulation of pro-inflammatory cytokines, chemokines, and proteases, which further perpetuate anabolic and catabolic activity, leading to the destruction of articular cartilage [7,8,9,10,11,12].

Articular cartilage is a highly specialized connective tissue that encircles the epiphysis of diarthrodial joints and is aptly designed to withstand compressive loads and high shear forces, enabling locomotion [1]. This distinct subtype of hyaline cartilage features a unique architecture comprising a highly organized extracellular matrix (ECM) and cells [1]. The ECM serves as the structural foundation for the gliding and cushioning functions of articular cartilage within a synovial joint complex; moreover, it acts as a signaling scaffold to preserve the phenotypic stability of chondrocytes. Consequently, any damage to the integrity of articular cartilage jeopardizes functional stability and alters quiescent phase and metabolic activity of non-renewable chondrocytes [12,13]. Over the past decades, research has primarily focused on chondrocytes as the sole cell type, leading to insufficient exploration of how pathogenesis affects the potency characteristics of other intrinsic cell niches, specifically mesenchymal progenitor cells (MPCs), in the context of joint diseases such as OA or RA.

The MPCs in articular cartilage affected by rheumatoid arthritis (RA) may be fundamentally different from those in osteoarthritis (OA) because of the autoimmune, inflammatory environment, which may alter resident cells. In RA, continuous exposure to pro-inflammatory cytokines (such as TNF-α, IL-1β, and IL-6) and immune pathways, such as Th1/Th2 signaling, may lead to prolonged transcriptional reprogramming, immune activation, and antigen-presentation signatures, which are largely absent or less pronounced in OA [14].

The conservative approaches aimed at reviving the intrinsic repair mechanisms using indigenous resident cells, such as chondrocytes or MPCs, to restore eroded articular cartilage are in vain, chiefly owing to the complexity of its avascularity, aging, and disease-driven malformations, and their scarcity. Consequently, therapies utilizing exogenous strategies evolved, particularly cell-based therapies that employed autologous, minimally modified, in vitro-cultured chondrocytes or bone marrow-derived mesenchymal stem cells (BM-MSCs) or MSCs sourced from various tissues to repair compromised articular cartilage [14,15]. Despite generally positive post-prognosis outcomes, the quality of neocartilage formed remains questionable, as histological techniques reveal its structure is partly constituted of fibrocartilage rather than hyaline cartilage [14,15,16].

Cartilage-derived mesenchymal progenitor cells (MPCs) represent a mechanistically distinct intermediate cell niche bridging classical MSCs and mature chondrocytes. Unlike MSCs, MPCs exhibit tissue-specific lineage priming with enhanced chondrogenic bias, reduced hypertrophy, and distinct TGF-β/SMAD signaling. In comparison to terminally differentiated chondrocytes, they retain proliferative, migratory, and multipotent capacities [14,16].

To achieve homotypic cartilage restoration, it is pivotal to enrich the cell source with chondroprogenitor cells (MPCs); recent findings have shown that MPCs exhibit superior chondrogenic potential compared to other cell types. MPCs are multipotent mesenchymal stem cells firmly committed to chondrogenic differentiation. So far, no efficient phenotypic markers have been identified to specifically define MPCs; they have primarily been characterized based on adhesion, colony-forming ability, and plasticity. Contrarily, it has been observed that differentiated chondrocytes cultured ex vivo will dedifferentiate and acquire fibroblast-like morphology. Additionally, due to the limited studies investigating the efficacy of MPCs in OA and RA, the current study aimed to identify MPCs using various combinations of phenotypic markers and to compare the potency characteristics of OA-MPCs and RA-MPCs, while adhering to the ISCT criteria.

## 2. Results

### 2.1. Morphology and Cellular Features of MPCs Derived from OA and RA Samples

**Figure 1:**
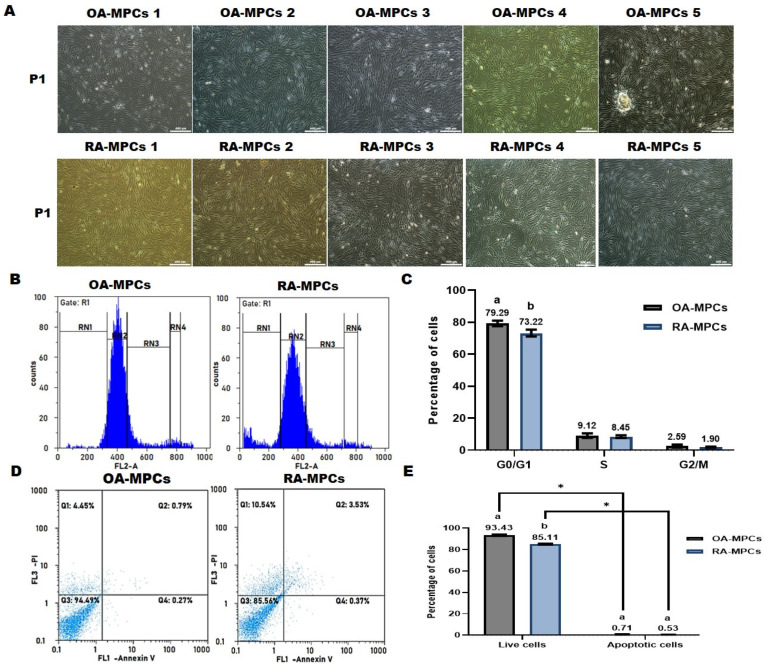
**Morphology, cell cycle status analysis, and apoptosis detection of mesenchymal progenitor cells (MPCs) isolated from osteoarthritis and rheumatoid arthritis cartilage.** (**A**) Plastic adherent MPCs exhibited a small spindle-shaped fibroblast-like morphology in osteoarthritis (OA-MPCs) and rheumatoid arthritis (RA-MPCs) cartilage-derived cell lines. The MPCs continued to display similar features until later passages P5. (**B**) Histograms indicating the OA-MPCs and RA-MPCs in different stages of cell replication. (**C**) The bar graph demonstrates the mean percentage of OA-MPCs and RA-MPCs under various stages of cell duplication. The analysis showed that more than 75% of MPCs were in the interphase of the cell cycle, with a significant difference between OA and RA (*p* < 0.05). (**D**) Histograms indicating the percentage of apoptotic MPCs in OA and RA cell lines. (**E**) Comparison of mean percentage of apoptotic OA-MPCs and RA-MPCs. The analysis showed less than 1% apoptotic MPCs, regardless of OA or RA. However, there was a significant difference (* *p* < 0.05) in the mean percentage of live cells in OA-MPCs and RA-MPCs. Image magnification 400 µm. Superscripts a and b above any two adjacent bars imply a statistically significant difference set at <0.05 (*p* < 0.05).

From collected tissue samples OA and RA (*n* = 5 each), primary explant cultures (P0) were successfully established, with the resulting cell lines referred to as OA-MPCs (*n* = 5) and RA-MPCs (*n* = 5). Wherein the release of OA-MPCs and RA-MPCs was apparent only after four to five days in culture (P0). From their onset in culture, MPCs displayed plastic adherence and fibroblast-like small spindle-shaped morphology with abundant agranular cytoplasm and prominent nuclei; further, similar features were evident till late passages (P5) in both the MPCs without any contrariness (Figure 1A). The cell cycle progression analysis of MPCs carried out at passage P3 depicted more than 75% of MPCs in the interphase (G0/G1) of the cell cycle with a significant difference between OA and RA (*p* < 0.05) (Figure 1B). The results, in turn, indicated that the majority of mitotically quiescent MPCs were present in both OA and RA. The remaining 25% of cells were distributed in other stages (S or G2/M) of cell duplication without any significant differences (*p* > 0.05) between OA and RA-MPCs (Figure 1C). The proportion of apoptotic cells among the MPCs at passage 5 (P5) from both disease states was minimal, at less than 1% (Figure 1D). Apoptosis analysis revealed a high percentage (>85%) of viable cells, showing a significantly greater (*p* < 0.05) number in OA (93.43%) compared to RA (86.11%) (Figure 1E).

### 2.2. Growth Kinetics, Cellular Age, and Pluripotency of OA- and RA-MPCs

**Figure 2:**
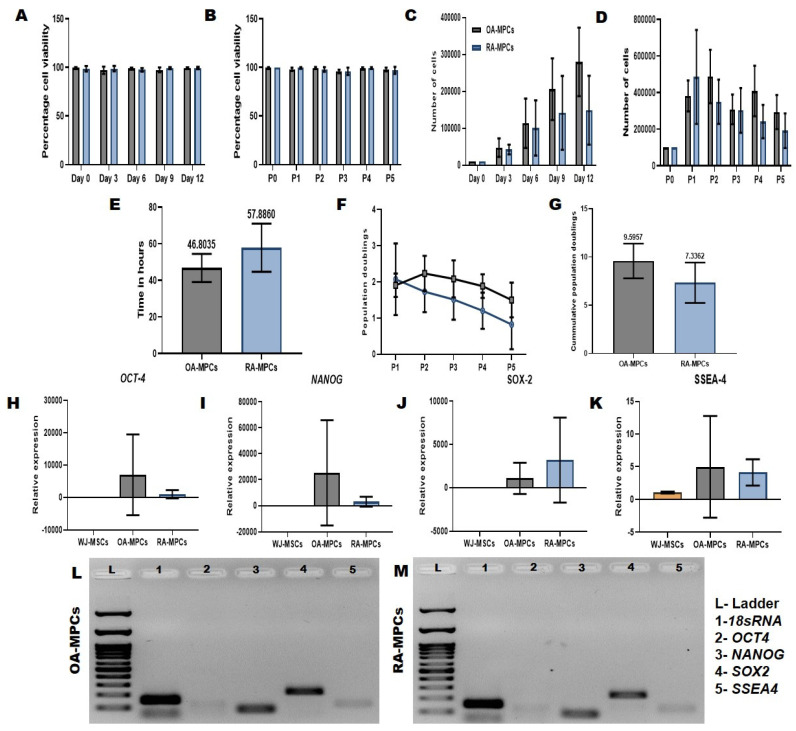
**Growth kinetics, cellular age assessment and pluripotency of OA-MPCs and RA-MPCs.** (**A**,**B**) Average percentage cell viability comparisons between OA-MPCs and RA-MPCs. MPCs, irrespective of their origins, depicted more than 95% of viability at different time points of a particular passage, P2, or between the passages, P0-P5, without any significant differences (*p* > 0.05). (**C**,**D**) Comparison of average growth potential amongst OA-MPCs and RA-MPCs within passage 2 and between passages P0 and P5. No significant differences (*p* > 0.05) were observed in the overall growth potential between OA and RA MPCs. (**E**) Comparative mean population doubling time (PDT) of OA-MPCs and RA-MPCs. Without any significant differences (*p* > 0.05), the overall PDT of OA-MPCs was 46.80 h, and that of RA-MPCs was 57.89 h. (**F**,**G**) Cellular age assessment of MPCs in terms of population doublings (PD), and cumulative population doublings (CPD). The PD decreased gradually from passage P1 to P5 in both OA-MPCs and RA-MPCs, without a significant difference (*p* > 0.05). The average CPD in OA-MPCs was 9.59, and that of RA-MPCs was 7.33. (**H**–**K**) Histograms representing the pluripotency genes (*OCT-4*, *NANOG*, *SOX-2*, and *SSEA-4*) expression in OA-MPCs and RA-MPCs. WJ-MSCs were used as the control, and *18sRNA* was used as a housekeeping gene. (**L**,**M**) The agarose gel images confirmed the expression of pluripotency genes.

When the overall percentage viability of OA-MPCs and RA-MPCs was evaluated on designated days in culture (Figure 2A) or during successive propagations from P0 to P5 (Figure 2B), cells displayed over 95% viability without any significant differences (*p* > 0.05) regardless of disease conditions. The proliferation rate measured at regular preset intervals during passage P2 showed a linear growth curve, beginning with an initial stagnant phase from day 0 to day 3, followed by an exponential phase in which the cells continued to proliferate, ultimately reaching a stationary phase by day 12. On day 12, the culture dishes also achieved complete confluency (Figure 2C). The proliferation rate examined between consecutive passages P0 to P5 revealed a sigmoid growth curve, starting with an initial lag phase at passage P0, followed by a logarithmic phase at passages P1 and P2 where cell numbers increased exponentially; after that, the cells entered a stagnant phase marked by a sharp decline in cell counts between passages P3 to P5. The statistically insignificant (*p* > 0.05) analogous frequency of overall growth rate was seen in both OA-MPCs and RA-MPCs (Figure 2D).

The overall PDT of OA-MPCs was 46.80 h, and that of RA-MPCs was 57.89 h with no significant (*p* > 0.05) differences (Figure 2E). The cellular age of MPCs, corresponding to population doublings, diminished with progress through subsequent passages from P0 to P5. The number of population doublings (PD) was directly proportional to the growth rate of MPCs from P0 to P5. The MPCs showed higher PD during passage P1 or P2, followed by a gradual decline (Figure 2F). The overall average cumulative population doublings (CPD) for OA-MPCs were 9.6, while for RA-MPCs, it was 7.3 (Figure 2G). No obvious differences in overall cellular age were observed between OA-MPCs and RA-MPCs. MPCs derived from both OA and RA displayed mRNA expression of pluripotency markers, including *OCT4*, *NANOG*, *SOX2*, and *SSEA4* (Figure 2H–K), which was also validated by 1.5% agarose gel electrophoresis (Figure 2L,M). The relative expression levels were compared with those of Wharton jelly-derived MSCs, which served as a control. There were no significant differences (*p* < 0.05) in the expression patterns of the panel of pluripotency transcription factors between OA-MPCs and RA-MPCs.

### 2.3. Colony-Forming Ability (CFA) and Alkaline Phosphatase (ALP) Activity of OA-MPCs and RA-MPCs

**Figure 3:**
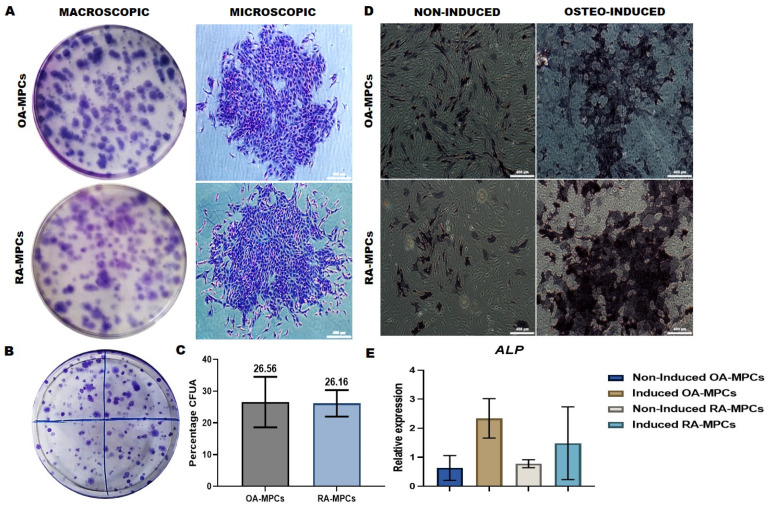
**Colony-forming ability (CFA) and alkaline phosphatase (ALP) activity of OA-MPCs and RA-MPCs.** (**A**) The MPCs, irrespective of their origin, could form colonies within 14 days in the culture. The colonies were positive for crystal violet staining. Further, the stained individual colonies were evident macroscopically and microscopically. (**B**) Representative image of the crystal violet-stained culture dish depicting the individual MPCs colonies used for scoring in the calculation of percentage CFA. The MPCs colonies that were positive for crystal violet staining and contained more than 50 cells were only considered for scoring. (**C**) The percentage CFA of OA-MPCs was 26.56% and that of RA-MPCs was 26.16% without any statistically significant difference (*p* > 0.05). (**D**) OA-MPCs and RA-MPCs on the 11th day of culture could form purple cytoplasmic coloration due to positive ALP staining, irrespective of their differentiated status, yet ALP activity was higher after osteogenic induction of MPCs for the same duration. (**E**) The relative mRNA expression analysis of *ALP* further supported the qualitative ALP staining. Image magnification 400 µm.

The OA- and RA-MPCs successfully formed colonies after 14 days of culture, demonstrating a homogeneous population of clonal cells as assessed by macroscopic and microscopic analyses (Figure 3A,B). Microscopic colonies that contained >50 cells, as verified through crystal violet staining, were deemed positive. Macroscopic assessment further indicated an average colony-forming ability (CFU) of 26.56% for OA-MPCs and 26.16% for RA-MPCs, with no significant (*p* < 0.05) differences (Figure 3C). On the 12th day of culture, MPCs demonstrated positive ALP staining, highlighting their inherent stemness, pluripotency, and differentiation potential (Figure 3D). Additionally, the ALP staining in MPCs displayed distinct morphological changes in osteogenesis-induced OA and RA-MPCs compared to the non-induced MPCs. The presence of ALP enzyme in the cytoplasm of MPCs was indicated by the dark purple insoluble staining observed in all induced and non-induced OA and RA cell lines. Furthermore, the relative expression of *ALP* in OA-MPCs and RA-MPCs further supported the qualitative detection (Figure 3E).

### 2.4. Senescence-Associated β-Galactosidase (SA β-Gal) Activity, Expression of Cellular Proliferation and Senescence Markers, and Cytogenetic Stability of OA-MPCs and RA-MPCs

**Figure 4:**
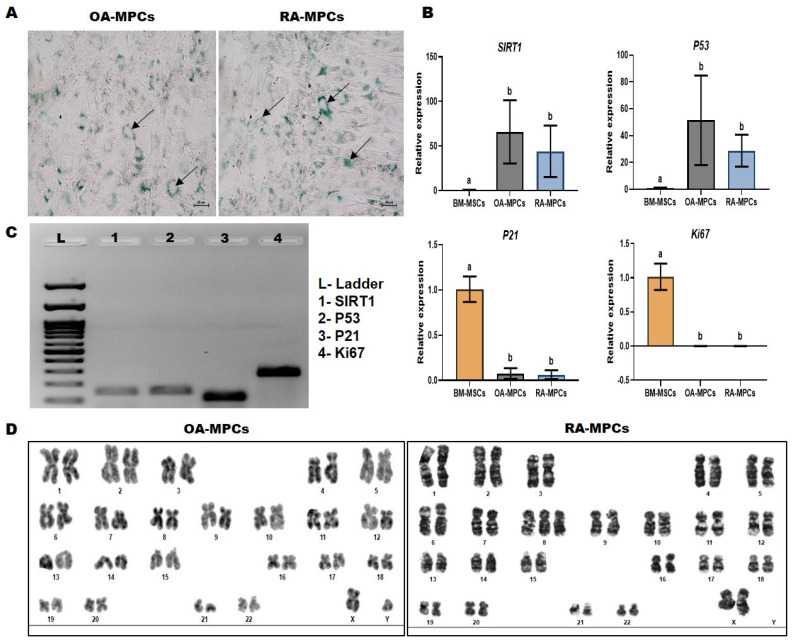
**Senescence-associated β-galactosidase (SA β-Gal) activity, mRNA expression of markers of cellular proliferation and senescence, and cytogenetic stability of OA-MPCs and RA-MPCs were assessed at passage 5.** (**A**) The SA β-Gal staining of OA-MPCs and RA-MPCs at passage 5 (P5) showed the bluish cytoplasmic staining, indicating senescent MPCs (arrows). (**B**) The relative expression of markers of cell proliferation (*SIRT1* and *Ki67*) and cellular senescence (*P53* and *P21*) in OA and RA MPCs, where BM-MSCs at P5 were used as an assay control and *18sRNA* was used as a housekeeping gene. (**C**) The agarose gel electrophoresis image confirming the cycling conditions and expression of the panel of markers in BM-MSCs. (**D**) The karyotypes of OA-MPCs and RA-MPCs acquired using G-banding were stable with twenty-two pairs of autosomes and a single pair of allosomes. Image magnification 50 µm. Superscripts a and b above any two adjacent bars imply a statistically significant difference, considered with *p* < 0.05.

Replicative senescence evaluated through senescence-associated β-galactosidase staining of MPCs showed that there were very few senescent cells present at passage P5 in both OA and RA-MPCs (Figure 4A). The mRNA levels of cell proliferation regulator markers (*SIRT1* and *Ki67*) and cellular senescence markers (P53 and P21) in OA-MPCs (*n* = 5) and RA-MPCs (*n* = 5) at passage 5 were examined through RT-qPCR. BMSCs at passage 5 served as the control calibrator for this assay. Compared with the control, significantly higher (*p* < 0.05) fold increases in *SIRT1* and *P53* expression were observed in both OA-MPCs and RA-MPCs, with no significant differences (*p* > 0.05) between the groups (Figure 4B). The levels of *P21* and *Ki67* were found to be the least in both OA and RA MPCs. The mRNA expression of the panel of markers did not show notable differences in expression patterns between OA and RA (Figure 4B). The primer sequences and cycling conditions for the transcription factors were standardized, and their expression in BMSCs was validated by agarose gel electrophoresis (Figure 4C). Karyotyping of metaphase spreads from MPCs collected at passage P3 using GTG banding demonstrated a normal chromosome count with clear morphology, consisting of 22 pairs of autosomes and 1 pair of sex chromosomes (Figure 4D). The karyotype analysis was conducted on at least 10 metaphase spreads for each of the OA and RA-MPCs. No differences in centromere appearance, size, or position, or in chromosome banding patterns, were noted in either OA-MPCs or RA-MPCs, confirming the cytogenetic stability of both cell types.

### 2.5. Immunophenotyping of OA-MPCs and RA-MPCs

**Figure 5:**
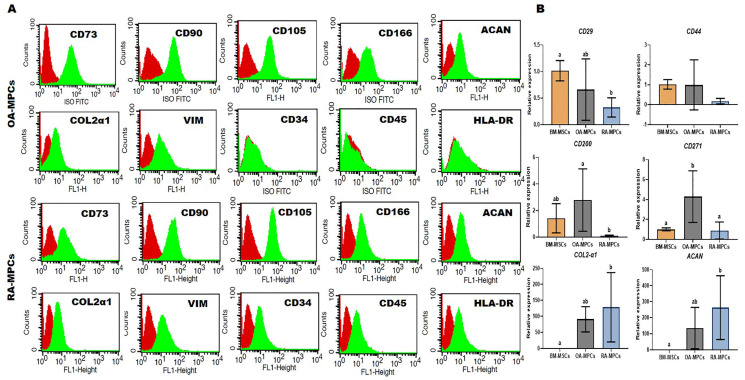
**Immunophenotyping of OA-MPCs and RA-MPCs by flow cytometry analysis and mRNA expression of selective phenotypic markers by RT-qPCR.** (**A**) Representative histograms depicting the expression of phenotypic markers, where the red-filled curve represents the respective isotype control and the green-filled curve indicates the expression of a specific marker as indicated. A total of 10,000 FITC-labeled cells were acquired and analyzed in duplicates. OA- and RA-MPCs were positive for mesenchymal lineage markers (CD73 and CD90), mesenchymal progenitor markers (CD105 and CD166), and the differentiated chondrocyte markers (ACAN, COL2α1, and VIM) and were negative for hematopoietic markers (CD34, CD45, and HLA-DR). (**B**) The bar graphs illustrate the mRNA expression of lineage-specific phenotypic markers. The OA- and RA-MPCs expressed the mesenchymal lineage markers (*CD29* and *CD44*), mesenchymal progenitor markers (*CD200* and *CD271*), and the differentiated chondrocyte markers (*COL2-α1* and *ACAN*). BMSCs were used as a control. Superscripts a and b above any two adjacent bars indicate the statistically significant difference (*p* < 0.05).

Flow cytometry analysis was performed to evaluate and confirm the stemness of both OA-MPCs and RA-MPCs (Figure 5A), and the data showed a distinct positive expression of MSC markers, such as CD73 and CD90, along with MPC markers, CD105, and CD166. Additionally, there was moderate positive expression of the differentiated chondrocyte markers ACAN and COL2α1, indicating their presence. Conversely, the negative expression of hematopoietic stem cell markers, including CD34, CD45, and HLA-DR, confirmed their absence in the cultures. The analysis of mean expression patterns of a section of CD markers demonstrated no statistically significant differences (*p* > 0.05) between OA-MPCs and RA-MPCs. Both the cells showed positive mRNA expression of mesenchymal lineage markers (CD29 and CD44), progenitor markers (CD200 and CD271), and differentiated chondrocyte markers (COL2-α1 and ACAN). The expression levels of mesenchymal lineage markers (CD29 and CD44), along with progenitor markers (CD200 and CD271), were similar to those of the control calibrator, bone marrow-derived MSCs (BM-MSCs), with no significant (*p* > 0.05) differences (Figure 5B). The fold expression of chondrocyte markers (COL2-α1 and ACAN) was notably higher in both OA and RA-MPCs compared to BMSCs. OA-MPCs exhibited a significantly greater expression of progenitor markers than RA-MPCs.

**Figure 6:**
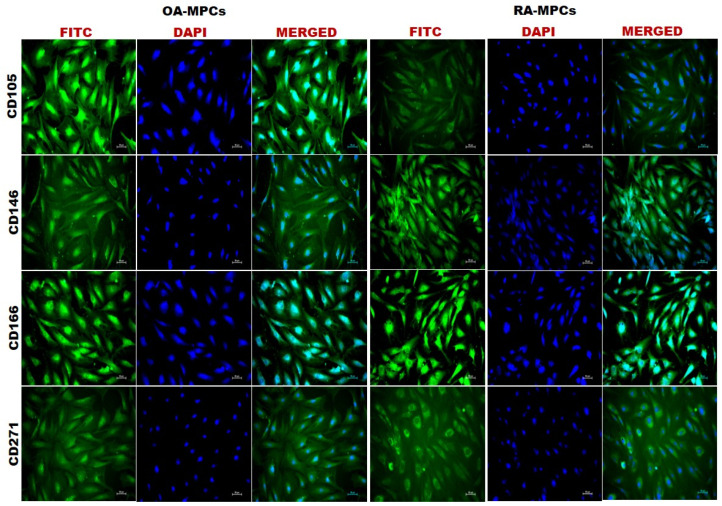
**Markers expression in OA-MPCs and RA-MPCs by immunofluorescence analysis.** Post-immunotagging with selected mesenchymal progenitor cell markers (CD105, CD146, CD166, and CD271) and counterstaining with DAPI, fluorescence microscopy confirmed the MPCs’ positive expression of these markers. Image magnification 50 µm.

Immunofluorescence analysis of MPCs, stained with a panel of markers (CD105, CD146, CD166, and CD271), confirmed the presence of all markers in both OA-MPCs and RA-MPCs. Upon careful microscopic observation, the fluorophores attached to the specific antigens on the cell surface and within the cytoplasm were apparent, alongside the DAPI-stained nuclei. These findings confirmed the existence of MPCs within the analyzed cell lines (Figure 6).

### 2.6. Adipogenic and Osteogenic Differentiation of OA-MPCs and RA-MPCs, and the mRNA Expression of Lineage-Specific Markers

**Figure 7:**
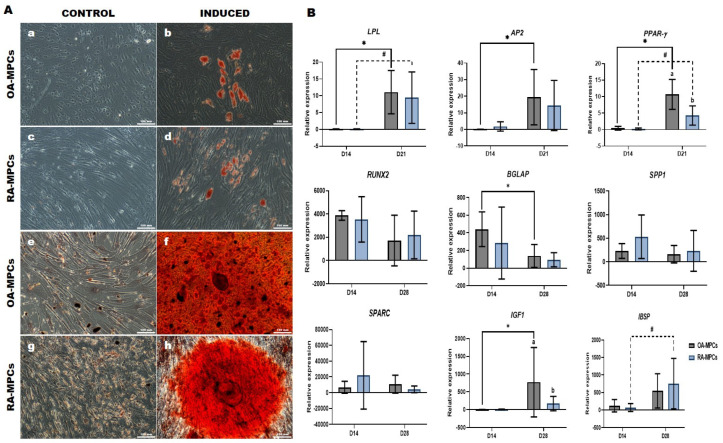
**Adipogenic and osteogenic differentiation of OA-MPCs and RA-MPCs and the mRNA expression of lineage specific markers.** (**A**) Adipogenic differentiation (**a**–**d**) and osteogenic differentiation (**e**–**h**) as confirmed through qualitative staining. MPCs were induced to differentiate into adipocytes by culturing them in adipogenic-specific media for 21 days. Following which they were positive for oil red o staining, wherein the lipid-filled cytoplasmic vacuoles were stained with red color, confirming the adipogenesis (**a**–**d**). MPCs were also capable of differentiating into osteocytes upon culturing them with specific induction media for 28 days. After which, osteogenic differentiation was confirmed by Alizarin red S (ARS) staining. Post-staining macroscopic and microscopic observations revealed deep brick-red precipitation of Ca^2+^ ions, resulting from the formation of the ARS-calcium chelating complex. The results further confirmed the mineralization in the extracellular matrix secreted by functional osteocytes (**e**–**h**). Image magnification-4x or 10x. (**B**) mRNA expression of adipogenic (*LPL*, *AP2*, and *PPAR-γ*) and osteogenic (*RUNX2*, *BGLAP*, *SPP1*, *SPARC*, *IGF1*, and *IBSP*) lineage specific markers as validated through RT-qPCR. mRNA expression of the markers of adipogenesis was analyzed on the 14th and 21st day in the specific induction media, and that of osteogenic lineage markers was analyzed on the 14th and 28th day in the specific induction media. Non-induced MPCs in the basal media cultured until specific time points were used as control, and *18sRNA* and *β-Actin* were used as housekeeping genes for adipogenic and osteogenic markers, respectively. Superscripts a and b above any two adjacent bars imply a statistically significant difference at *p* < 0.05. * *p* < 0.05, # *p* < 0.05.

The adipogenic induction lasting 21 days led to the noticeable presence of signet-shaped lipid-filled cytoplasmic vacuoles in both OA-MPCs and RA-MPCs. These structures, when stained with Oil Red O, produced a water-insoluble red coloration within the cytoplasm of MPCs, confirming adipogenesis (Figure 7A). The induction of adipogenesis in OA-MPCs and RA-MPCs was further validated by assessing mRNA expression levels of lineage-specific transcription factors such as *LPL*, *AP2*, and *PPAR-γ* on the 14th and 21st days of the induction period (Figure 7B). The relative mRNA expression of all the examined genes was found to be lower at 14 days of induction, while a significant (*p* < 0.05) increase was observed at the conclusion of the 21-day induction. Although no significant (*p* > 0.05) differences were found between OA-MPCs and RA-MPCs regarding the overall expression of *LPL* and *AP2*, the expression of *PPAR-γ* was notably higher in OA-MPCs after 21 days of induction compared to RA-MPCs.

At the conclusion of the 28th day, the osteogenic cell cultures exhibited changes in morphology and extracellular matrix deposition under a microscope. In contrast, the non-induced cultures in basal growth media maintained a spindle-shaped, fibroblast-like appearance throughout the culture (Figure 7A). Additionally, Alizarin Red S (ARS) staining of the mineral nodules formed highlighted the ARS-calcium chelating complex, characterized by deep brick-red precipitations of Ca^2+^ ions, which were observable both macroscopically and microscopically. All analyzed OA-MPCs and RA-MPCs showed positive results for ARS staining. Moreover, mRNA expression analysis of osteogenic lineage-specific genes, including *RUNX2*, *BGLAP*, *SPP1*, *SPARC*, *IGF1*, and *IBSP*, on the 14th and 28th days of the induction period confirmed the osteogenic differentiation of OA-MPCs and RA-MPCs (Figure 7B). Overall, the mRNA expression analysis indicated no significant (*p* > 0.05) differences between OA-MPCs and RA-MPCs.

### 2.7. Chondrogenic Differentiation of OA-MPCs and RA-MPCs and mRNA Expression of Lineage-Specific Markers

**Figure 8:**
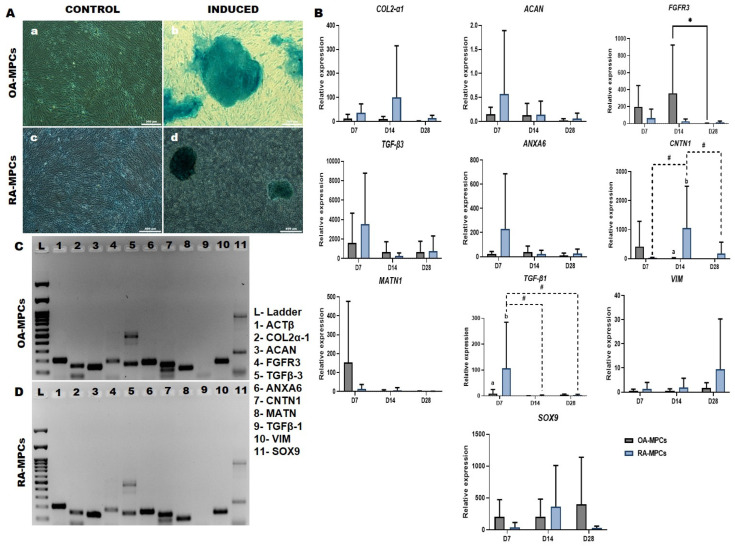
**Chondrogenic differentiation of OA-MPCs and RA-MPCs and the mRNA expression of lineage-specific markers.** (**A**) (**a**,**c**) Non-induced OA-MPCs and RA-MPCs. (**b**,**d**) Chondrogenic differentiation was confirmed through qualitative staining. MPCs were induced to differentiate into chondrocytes by culturing them in specific induction media for 28 days. Following this, they were positive for Alcian blue staining, wherein the acid mucins secreted and deposited by the functional chondrocytes as spheroids in the extracellular matrix were stained with blue color, confirming chondrogenesis. Image magnification-4x or 10x. (**B**) mRNA expression of chondrogenic (*COL2-α1*, *ACAN*, *FGFR3*, *TGF-β3*, *ANXA6*, *CNTN1*, *MATN1*, *TGF-β1*, *VIM*, and *SOX9*) lineage specific markers as validated through RT-qPCR. mRNA expression of the panel of markers was analyzed on 14th and 28th day in the specific induction media. Non-induced MPCs in the basal media cultured until specific time points were used as control, and β-Actin was used as a housekeeping gene. (**C**,**D**) Photomicrographs of agarose gel electrophoresis of the panel of markers validating chondrogenesis and the cycling condition by producing bands at specific base pairs. Superscripts a and b above any two adjacent bars imply a statistically significant difference measured at a confidence interval of 95% (*p* < 0.05). * *p* < 0.05, # *p* < 0.05.

The chondrogenic potential of OA-MPCs and RA-MPCs was initially assessed by 2D monolayer differentiation, which transformed the cells into chondrocytes. Following the induction period, microscopic examination of the chondrogenesis-induced OA and RA-MPCs showed the formation of a multilayered cell sheet featuring distinct spheroids that are abundant in mucopolysaccharide and sialomucins, which are key components of the cartilage extracellular matrix. Furthermore, Alcian blue staining indicated that the spheroids contained glycosaminoglycans and glycoproteins (Figure 8A). When the monolayer differentiated OA-MPCs and RA-MPCs were assessed for mRNA expression of chondrogenic lineage markers, such as *COL2α-1*, *ACAN*, *FGFR3*, *TGFβ-3*, *ANXA6*, *CNTN1*, *MATN*, *TGFβ-1*, *VIM*, and *SOX9* on days 7, 14, and 28 of culture, the results confirmed positive expression of all the markers. Prior to the RT-qPCR analysis, the optimal working conditions for all gene panels were evaluated through agarose gel electrophoresis (Figure 8C,D). No significant (*p* > 0.05) differences were detected in the expression levels of the marker panel between OA-MPCs and RA-MPCs.

**Figure 9:**
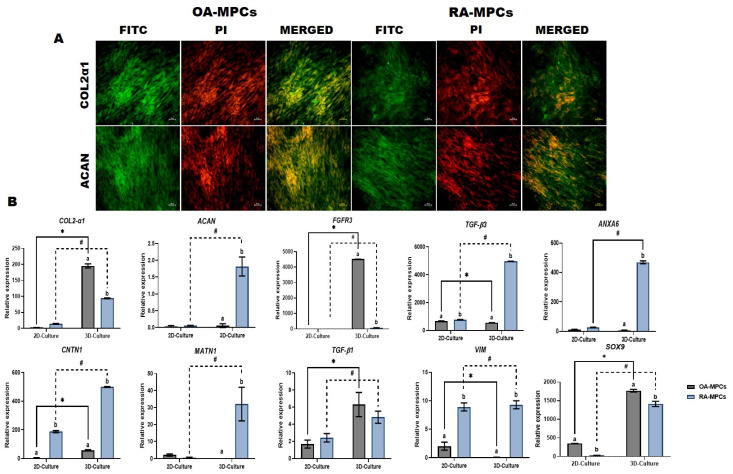
**Chondrogenic differentiation of OA-MPCs and RA-MPCs in 3-Dimensional (3D) mass culture and mRNA expression of the panel markers in 2-Dimentional (2D) monolayer culture Vs 3D pellet culture.** (**A**) The microphotographs show immunostaining of 3D mass-cultured MPCs with chondrocyte-specific markers (COL2-α1 and ACAN). MPCs were induced to differentiate into chondrocytes under 3D mass cultures nourished with specific induction media for 28 days. After which, they were stained with chondrocyte markers and counterstained with DAPI. Following this, fluorescence microscopy confirmed the fluorophores’ emission, indicating selective marker binding and expression. Image magnification 50 µm. (**B**) mRNA expression of chondrogenic lineage specific markers (*COL2-α1*, *ACAN*, *FGFR3*, *TGF-β3*, *ANXA6*, *CNTN1*, *MATN1*, *TGF-β1*, *VIM*, and *SOX9*) in 2D monolayer culture Vs 3D pellet culture. MPCs were induced to differentiate into chondrocytes by nourishing them with specific induction media for 28 days in 2D monolayer and 3D pellet cultures. Followed by the mRNA expression and comparison, the relative fold expression of a panel of selected markers was higher in 3D pellet cultures than in 2D cultures. Superscripts a and b above any two adjacent bars imply a statistically significant difference measured at a confidence interval of 95% (*p* < 0.05). * *p* < 0.05, # *p* < 0.05.

Furthermore, the high-density mass-cultured OA-MPCs and RA-MPCs displayed positive results for the immunostaining of chondrocyte-specific markers, such as *COL2α-1* and *ACAN*, as confirmed by fluorescence microscopy (Figure 9A). The chondrogenic potential of monolayer (2D) cultured and pellet (3D) cultured OA-MPCs and RA-MPCs was assessed through mRNA expression using RT-qPCR. The set of chondrogenic lineage markers evaluated included *COL2α-1*, *ACAN*, *FGFR3*, *TGFβ-3*, *ANXA6*, *CNTN1*, *MATN*, *TGFβ-1*, *VIM*, and *SOX9* (Figure 9B). The fold expression of all genes, except *ACAN*, *MATN*, and *ANXA6*, significantly increased (*p* < 0.05) in the 3D pellet culture. The relative expression levels of *ACAN*, *MATN*, and *ANXA6* showed a significant (*p* < 0.05) increase only in the 3D-cultured RA-MPCs, but not in the 3D-cultured OA-MPCs. The relative fold changes in the analyzed genes varied between 3D-cultured OA-MPCs and RA-MPCs (Figure 9B).

## 3. Discussion

Chondroprogenitors, although classified as mesenchymal stem cells (MSCs), serve as intermediaries between bone marrow-derived MSCs (BM-MSCs) and chondrocytes due to their similar potency and expression of epitope markers [16,17,18,19,20]. Over time, multipotent progenitor cells (MPCs) have been viewed as a potentially effective alternative source for cartilage repair compared to conventional sources like chondrocytes or BM-MSCs, primarily because of their enhanced chondrogenic potential and resistance to hypertrophy [16]. Chondrocytes were previously thought to be the sole cell type; extensive research has focused on them. Consequently, the effect of pathogenesis on the potency of MPCs is yet to be ascertained. Nevertheless, this study is among the few to examine and identify a readily accessible niche of MPCs in cartilage from patients with osteoarthritis (OA) and rheumatoid arthritis (RA).

From the onset of primary cultures, MPCs from OA and RA have displayed plastic-adherent, small, spindle-shaped morphology. This appearance is similar to that of traditional MSC sources [2,16,20,21]. MPCs are recognized as a quiescent niche with varying regenerative capabilities depending on the species, organ, or developmental stage. Cell replacement and regeneration can occur in two circumstances: during normal tissue maintenance to replenish depleted cells, and in response to external trauma, injury, or amputation. Disease states can trigger rapid cell proliferation, facilitated by a significantly altered cell cycle that enables cells to transition swiftly from DNA synthesis to division while minimizing the duration spent in the intervening gap phases [22]. Annexin V-based analyses provide the most reliable means of assessing plasma membrane asymmetry loss [23]. Since cell cycle dynamics and cellular apoptosis are indicators of cell quality, these assessments were performed to determine whether OA or RA pathogenesis has disrupted MPC homeostasis.

The unaltered morphology, viability, and sustained growth kinetics through successive passages are crucial for their application in tissue engineering. Previous literature has indicated that chondrocytes are terminally differentiated and thus cannot proliferate or differentiate [24]. The present study shows a steady proliferation rate and higher viability that persisted until later passages (P5). The cellular age is often indicated by the number of passages a cell has undergone. However, this measure can be imprecise due to differences in initial cell seeding density across passages. To tackle this issue, it is widely accepted to use population doublings (PD) or cumulative population doublings (CPD) to monitor cellular age in vitro. In this study, the PD values for both OA-MPCs and RA-MPCs declined as passage progressed from P1 to P5, consistent with previously documented population doublings of MSCs from multiple sources [25].

The moderate growth kinetics observed in MPCs, while not markedly different between OA and RA, were further confirmed by significantly elevated (*p* < 0.05) SIRT1 mRNA expression levels. *SIRT1*, an NAD+-dependent deacetylase, plays a crucial role in regulating longevity in mesenchymal stem cells (MSCs), which give rise to mesenchymal lineage tissues. Additionally, *SIRT1* has been shown to enhance MSC self-renewal capacity in response to intrinsic and extrinsic factors that contribute to DNA damage, reactive oxygen species, genomic instability, hyperinflammation, and apoptosis [26]. However, *Ki67* expression in both OA-MPCs and RA-MPCs did not differ significantly from that in control BMSCs. *Ki-67* is a cell proliferation marker and a commonly used prognostic marker for various cancers. Although *Ki67* is correlated with cell proliferation, its role remains controversial, as normal tissue-derived, viable, moderately proliferating cells have been reported to exhibit low or absent protein expression [27].

The present study findings illustrate the expression of *OCT4*, *SOX2*, *NANOG*, and *SSEA4* in both OA and RA MPCs. However, mRNA expression alone does not suffice to confirm their functional roles in the related signaling pathways unless the expression of the corresponding functional protein is established [20]. Additionally, these pluripotency markers inhibit the expression of differentiation-related transcription factors, thereby preserving stem cell pluripotency. MSCs are thought to be quiescent cells that, when prompted, can replicate into a stem cell clone and a transiently amplifying progenitor cell. Subsequently, this progenitor cell undergoes a limited number of divisions before ultimately differentiating into functional adult tissue cells [28]. The presence of these transcription factors in both OA-MPCs and RA-MPCs may indicate the undifferentiated status of transient progenitor cells, which may also correspond with the significant *ALP* activity observed in the MPCs, as ALP is another key marker used to confirm pluripotency [29,30,31].

The clonogenic assay, also known as the CFU assay, is an in vitro cell survival assay that evaluates cell survival based on a single cell’s capacity to form a colony, which requires a minimum of 50 cells. This assay effectively tests the entire cell population for its ability to undergo seemingly “unlimited” division, a characteristic that is only passed on to a subset of cells. In the diverse cell populations harboured in articular cartilage, mature chondrocytes typically do not possess clonogenic potential, although they express certain putative markers. In contrast, the colony-forming ability of MPCs distinguishes them from other cell types [6]. Thus, the ability to form colonies is a distinctive feature of stem/progenitor cells and can significantly aid in identifying MPCs.

The results inferred MPCs’ resistance to SA-β-Gal activity and cytogenetic instability, likely due to their relatively quiescent state and their non-involvement in the pathogenesis of cartilage. These findings were further supported by the lowest mRNA expression levels of senescence biomarkers, such as *P16*, *P21*, and *P53*. When contrasted with the control BMSCs, MPCs from OA and RA displayed higher mRNA expression levels of *P53* (*p* < 0.05) while exhibiting lower levels of *P16* and *P21* (*p* > 0.05). *P53* and *P21* not only mediate cellular senescence but also regulate cell cycle progression and apoptosis, which are typically activated in healthy cells to prevent tumor development. *P53* is often referred to as the “guardian of the genome,” as it plays a critical role in tumor suppression and initiates cell cycle arrest and apoptosis following DNA damage, thus maintaining genomic stability [32,33]. *P16* is involved in halting the cell cycle and inactivating cyclin D-cyclin-dependent kinases, leading to growth suppression and cell cycle arrest in the G1 phase. An increase in *P16* mRNA expression is associated with advanced aging in stem cells, which may subsequently diminish their self-renewal capacity [34].

While the specific surface markers indicating the MPCs population remain unidentified to this day, they have been indirectly characterized through their expansion in culture, as their defining phenotypic traits as progenitor or stem cells are not well established [35]. In this investigation, both OA-MPCs and RA-MPCs exhibited positive expression of MSC markers (CD73, CD90, and CD166), which were found to be negative for hematopoietic markers (CD34, CD45, and HLA-DR), thereby confirming their phenotypic characteristics in alignment with the ISCT position statement [17,19]. The analyzed progenitor cell markers (CD105, CD146, CD166, CD200, and CD271) represent one of the few thoroughly characterized marker combinations that may indicate their niches within articular cartilage. CD105, commonly referred to as endoglin, along with CD166, the activated leukocyte cell adhesion molecule, has been proposed as identifying MPCs niches that possess enhanced chondrogenic potential in both OA and normal cartilage [2,32,36]. Additionally, CD166+ progenitor cells were identified in the perichondrium, and elevated levels of CD146 (Melanoma cell adhesion molecule) have been noted in later stages of OA and suggested as potential MPC markers [37,38]. Interestingly, however, the MPCs were CD166+, and only a few studies have regarded CD146 as an indicator of chondroprogenitor cell subpopulations with greater chondrogenic potential and clonogenic ability [39,40]. CD200 and CD271 are recognized as MSC/chondroprogenitor niche markers associated with increased proliferation rates, enhanced chondrogenic potential, and reduced apoptosis [41,42].

Additionally, markers indicative of differentiated chondrocytes, such as *ACAN*, *COL2α1*, and *VIM* showed positive expression when analyzed at an early passage 3, suggesting the diversity in cartilage associated with OA and RA. It remains uncertain whether the intermediate filament vimentin (*VIM*), a crucial element of the chondrocyte cytoskeleton, serves as a marker for MPCs or for mature chondrocytes [43,44]. Nevertheless, *VIM*’s functional significance in regulating chondrogenic differentiation of MSCs from various tissue sources outside cartilage has been established [2,43]. Our research is the first to investigate *VIM* expression in MPCs derived from OA and RA cartilage, aiming to elucidate its role in chondrogenesis.

Plasticity is an exceptional trait of MSCs, enabling them to differentiate into osteocytes, adipocytes, and chondrocytes. In this study, MPCs in monolayer cultures could undergo osteogenesis, adipogenesis, and chondrogenesis when cultured in the appropriate medium.

The expression levels of transcription factors associated with osteogenesis, including *RUNX2*, *BGLAP*, *SPARC*, *ALP*, *IGF1*, *IGF2*, *IBSP*, *TGFβ-1*, *BMP2,* and *BMP6*, which determine how multipotent progenitor cells (MPCs) differentiate into osteoblasts or osteocytes, were found to be increased throughout the induction phase. *RUNX2* is the initiation factor necessary for the transition of multipotent mesenchymal stem cells into immature osteoblasts, although it subsequently inhibits the maturation of osteoblasts [45,46]. During osteoblast differentiation, *RUNX2* is expressed at lower levels in non-induced MSCs, but after osteogenic induction, its expression is enhanced in preosteoblasts, peaks in immature osteoblasts, and is then downregulated in mature osteoblasts [45,46,47]. *BGLAP* and *SPARC* are genes that encode for non-collagenous proteins that play crucial roles in osteogenesis [47]. These proteins are predominantly found in the bone extracellular matrix produced by osteoblasts. Additionally, *SPARC* is a non-structural glycoprotein that participates in cell–matrix interactions, exhibiting strong calcium binding and high affinity for collagen in bone [47,48,49]. *ALP* is the first marker to appear during the onset of mineralization, simultaneously indicating the differentiation from pre-osteoblasts to osteoblasts. This transcription factor, in its enzymatic form, is primarily associated with phosphate metabolism and matrix elaboration [50,51]. *IGF1* promotes bone development by fostering osteoblast proliferation and their differentiation into osteocytes. *IBSP* is thought to play a significant role in the initial mineralization of bone and is selectively expressed by differentiated osteoblasts [52]. Likewise, *IGF2* is a potential factor for bone development, repair, and remodeling, enhancing osteogenesis by modulating *RUNX2* and *COL1* [53]. *TGFβ-1, BMP2,* and *BMP6* are transcription factors that belong to the *TGF-β* superfamily. *TGF-β/BMP* is implicated in the vast majority of cellular processes, including bone formation and remodeling. The coordinated activity of *RUNX2* and *TGF-β/BMP* is critical for deriving a skeleton on which the bone formation or remodeling occurs [54].

In this study, MPCs exhibited intracellular accumulation of lipid droplets, indicating the adipocytic morphology, and it was further confirmed by the expression of adipocyte-specific genes, such as *LPL*, *AP2*, and *PPAR-γ*, which are the key up-regulators in lipid metabolism, lipid synthesis, and adipocyte differentiation [55].

CD105+/CD166+ cells do not express markers of differentiated chondrocytes, such as *ACAN*, *COL2-α1* [2]. Vimentin, although a marker of the cytoskeleton, is a regulator of chondrogenesis, as confirmed by a few studies showing that siRNA-mediated vimentin knockdown inhibited cartilage-specific ECM production [56]. However, MPCs, upon chondrogenic induction in monolayer cultures for 28 days, expressed chondrocyte markers (*COL2α-1*, *ACAN*, *FGFR3*, *TGFβ-3*, *ANXA6*, *CNTN1*, *MATN*, *TGFβ-1*, *VIM*, *SOX9*) and formed typical alcian blue-stained spheroids. MPCs derived from normal or OA articular cartilage and SVMSCs in both RA and OA conditions are previously reported to exhibit higher chondrogenic potential than adipogenesis or osteogenesis [2,57]. However, whether articular cartilage-derived later-stage OA-MPCs and RA-MPCs exhibit such comparable chondrogenic potential is yet to be ascertained. Further, the role of VIM in chondrogenesis was confirmed by siRNA-mediated knockdown, which inhibited cartilage-specific ECM production [58].

Along with well-studied lineage-specific markers, such as *ACAN*, *COL2-α1*, *SOX9*, *TGF-β3*, and *TGF-β1*, this study demonstrated the expression of molecular markers involved in chondrogenesis or chondrocyte proliferation, including *FGFR3*, *ANXA6*, *CNTN1*, *MATN*, and *VIM*. In addition to promoting chondrogenesis, the fibroblast growth factors (FGFs) are known to modulate and maintain various biological properties, including multilineage differentiation through *FGFR3*-mediated signaling [59]. Amongst them, *FGFR3* is a marker of mesenchymal pre-cartilaginous stem cells [59,60,61]. The role of *ANXA6* in cartilage-derived cells is unclear, even though it is highly expressed in articular chondrocytes compared to other types of MSCs. However, *ANXA6* is known to regulate and modify *NF-kB* and *Wnt/β-catenin* signaling pathways, the activation of which releases the *MMP-13* and leads to the degradation of articular cartilage [59]. It is established that the matrilins are the non-collagenous ECM proteins consisting of four members, among which *MATN1* and *MATN3* are specifically expressed in articular cartilage and growth plate [60]. The mRNA expression of these key markers further confirmed the chondrogenic potential of OA and RA cartilage-derived MPCs.

Although studies have long suggested that in vitro chondrogenic induction of adult human MSCs/MPCs in 2D monolayer cultures is sufficient to support cartilaginous tissue formation in vivo [62,63], with recent advancements, 3D micro-mass cultures with biomaterials are known to provide a suitable environment for the induction and maintenance of chondrogenic phenotypes [62,64,65,66]. The present study outcomes also indicate a relatively higher fold change in the expression of chondrogenesis regulator genes, further implying superior chondrogenesis.

Although the present study identified significant differences in the expression of pluripotency-, senescence-, and lineage-associated genes between OA-MPCs and RA-MPCs, these observations were based primarily on transcript-level analyses. While gene expression profiling provides important insights into disease-specific molecular characteristics, mRNA abundance may not always directly reflect protein expression or functional activity. In accordance with the minimal position statement recommended by the International Society for Cell & Gene Therapy, the current study focused on cellular phenotyping, plastic adherence, and trilineage differentiation, with RT-qPCR used for exploratory molecular assessment. Therefore, protein-level validation of key markers, such as pluripotency regulators, senescence-associated proteins, and differentiation-related factors through Western blotting, ELISA, or immunostaining would further strengthen the biological interpretation of the findings and should be addressed in future investigations.

## 4. Materials and Methods

### 4.1. Patients and Sample Collection

All the subjects involved in the study accorded prior written informed consent. Osteo-arthritis (OA) and Rheumatoid arthritis (RA) articular cartilage samples (*n* = 5 each) from distal femoral condyles adjacent to the site of lesions were collected from the patients who were presented with stiffness or tenderness in the knee joint due to grade 3 or grade 4 OA (Kellgren and Lawrence system for classification of OA knee) or patients presenting with chronic pain and discomfort in the knee joint, Also upon further examination of reviewing symptoms such as tenderness, stiffness, and unsteadiness, X-rays, and lab tests were confirmative of advanced rheumatoid arthritis (2010 American College of Rheumatology/European League Against Rheumatism Classification Criteria).

### 4.2. Ethics Approval and Consent to Participate

The present in vitro study design, including the experimental protocols, was reviewed and approved by the Central Ethics Committee of Nitte (Deemed to be University, Ref: NU/CEC/2018/0177/D). Written informed consent was obtained from the subjects prior to sample collection. Further, all experiments were performed in accordance with the Declaration of Helsinki, and also by following relevant guidelines and regulations.

### 4.3. Chemicals, Media, and Reagents

The chemicals used were purchased from Sigma Chemical Company (St. Louis, MO, USA) and cell culture media and reagents from Gibco-Invitrogen (Thermo Fisher Scientific, Life Technologies, Grand Island, NY, USA), unless otherwise specified.

### 4.4. Isolation and Propagation of MPCs

For the isolation of MPCs, collected cartilage tissue samples were brought into a class 2 A2 biosafety cabinet, washed twice in DPBS supplemented with 100 U/mL penicillin and 100 μg/mL streptomycin, and minced into small pieces using sterile surgical instruments. To facilitate the release of MPCs, they were partially digested enzymatically with 0.1% collagenase type II for 3 h at 37 °C in a humidified 5% CO_2_ incubator. Following this, semi-digested tissue pieces were seeded for explant culture onto 100 mm culture dishes pre-coated with 0.1X attachment factor (Gibco) overnight at 37 °C and supplemented with Dulbecco’s Modified Eagle Medium (DMEM/F12, 1:1) containing 20% fetal bovine serum (FBS), 1X glutamax, 0.1 mM mercaptoethanol, 1X non-essential amino acids (MEM-NEA) and 1X Pen-Strep. The cultures were maintained throughout the period under optimal culture conditions, including 37 °C in a humidified 5% CO_2_ incubator. Upon 70% confluency, Primary cultures (P0) were subpassaged up to the fifth passage (P5) supplemented with complete media containing 10% FBS, for further characterisation. All experiments were conducted before P5, with a minimum of 3 replicates.

### 4.5. Morphology, Cell Cycle Status Analysis, and Apoptosis Detection of MPCs

The cellular morphology of MPCs from the primary culture P0 through later sub passages P5, at different time intervals within a particular passage, was analysed and documented using a phase-contrast photo microscope (Olympus, Tokyo, Japan).

For cell cycle analysis, MPCs from the passage (P5), at a confluency of 70–80% were trypsinized, washed with DPBS, and fixed with 70% cold ethanol. Cells were then treated with 0.5 mg/mL RNase A (Himedia, Thane, India) and stained with 50 μg/mL propidium iodide (Invitrogen, Grand Island, NY, USA) in the dark at 37 °C for 30 min. Following this, the DNA content was determined by flow cytometry (BD FACS Calibur, Becton Dickinson, NJ, USA) and the percentage of cells in different phases of the cell cycle was assessed. A minimum of 10,000 events were acquired per sample.

The presence of apoptotic MPCs was assessed using an Annexin V apoptosis detection kit-FITC (eBioscience, Invitrogen, Grand Island, NY, USA) according to the manufacturer’s instructions. For the assay, MPCs at 70–80% confluency of passage 5 were detached using TrypLE E (Gibco, Grand Island, NY, USA), and if any FITC-conjugated Annexin V-bound cells were present, they were detected by flow cytometry (BD FACS Calibur). A minimum of 10,000 events were acquired per sample.

### 4.6. mRNA Analysis by RT-qPCR

Cells (passages P3 and P5) stored at −80 °C/liquid nitrogen were thawed, and total RNA isolation was performed using the RNAiso Plus kit (Takara, Tokyo, Japan) according to the manufacturer’s instructions. 500 ng of template RNA was transcribed into cDNA by using Prime Script RT Reagent kit (Takara, Japan) following the manufacturer’s instructions. The mRNA level was quantified by real-time PCR (Applied Biosystems, StepOnePlus™, Waltham, MA, USA) using SYBR^®^ Premix ExTaq™ II (Takara, Tokyo, Japan). The cycling conditions were 10 min of initial denaturation at 95 °C, 40 cycles of 94 °C for 30 s, 60 °C for 1 min, and 72 °C for 40 s. Final extension at 72 °C for 5 min, followed by a dissociation stage. The ΔΔCT method was used to evaluate the relative expression fold of target genes, normalised to β-actin or 18s RNA, for each sample. The target gene expression in the amplified products was confirmed by 1.5% agarose gel electrophoresis. The primer sequences of the genes are presented in Table 1.

Bone marrow-derived mesenchymal stem cells (BM-MSCs) used as the calibrator control for RT-qPCR analysis were established from bone marrow aspirates collected from independent donors after written informed consent was obtained, in accordance with institutional ethical committee approval. Mononuclear cells were isolated and cultured under standard conditions to establish BM-MSCs. Total RNA extracted from these BM-MSC cultures was used as the calibrator reference sample in comparative gene expression analysis (2^−ΔΔCt^ method) for OA-MPC and RA-MPC samples.

Growth kinetics, cellular age assessment, colony-forming ability (CFA), alkaline phosphatase (ALP) activity, and senescence-associated β-galactosidase (SA β-Gal) activity were assessed as described previously [21].

### 4.7. Cytogenetic Stability

Cytogenetic stability of MPCs was assessed at 70% confluency of passage 5 (P5) by GTG banding on metaphase spreads. When MPCs were presented with a sufficient number of doublets, confirmed post-observation under high-power magnification of a bright-field microscope, 50 µL of 10 µg/mL colcemid was added to the growth medium and incubated for 2 h at normal culture conditions. Afterward, the cells were harvested and incubated with pre-warmed 0.075 M KCL for 15 min at 37 °C. Then the hypotonic solution was neutralized by adding the cold Carnoy’s fixative (3:1 methanol and glacial acetic acid) dropwise. The cells were prefixed and stored in the Carnoy’s fixative at 4 °C until they were used for dropping.

The stored MPCs were rewashed with freshly prepared Carnoy’s fixative. Then ~20 µL of a concentrated cell suspension in Carnoy’s fixative was dropped onto a clean microscopic slide over the humidity chamber and air-dried at room temperature. Further, the slides were dried at 60 °C overnight in an oven before Giemsa staining. The slides were then dipped in trypsin solution and NaCl solutions for a few seconds in each and then stained with 1% Giemsa stain. Post washing the excess stain and air drying 10–15 finely banded metaphase spreads were identified and acquired using the bright field oil immersion (100x) of a fluorescence microscope (Olympus, Tokyo, Japan). Karyotyping was performed using the GenASIs software (Version 8.3, Applied Spectral Imaging, Ha’Emek, Israel). The results were carefully reviewed and recorded for any chromosomal abnormalities.

### 4.8. Immunophenotyping of MPCs by Flow-Cytometry Analysis and by Fluorescence Microscopy

MPCs at 70% confluency were harvested and fixed in 3.7% paraformaldehyde for 20 min. For intracellular marker expression, cells were permeabilized by 0.1% Triton X-100 for 20 min. Then, MPCs nonspecific antigens were blocked by incubating with 1% bovine serum albumin (BSA) for 30 min at room temperature. Followed by they were incubated with anti-human CD73 (Biolegend, San Diego, CA, USA, 1:100) CD90 (E-bioscience, San Diego, CA, USA, 1:100), CD105 (BD Pharmingen, BD Biosciences, San Jose, CA, USA, 1:20), CD146, CD166, ACAN, COL2α1, CD34 (Biolegend, 1:100), CD45 (E-bioscience, 1:100), HLA-DR (Biolegend, 1:100) at 4 °C overnight. Subsequently, the primary antibody-labeled MPCs were incubated in the dark with fluorescein isothiocyanate (FITC)-conjugated secondary antibody (E-Bioscience, 1:100) at 37 °C for 45 min. The standard was established by isotope-matched control (e-Bioscience). A total of 10,000 FITC-labelled cells were acquired and analyzed by flow cytometry (BD FACS Calibur).

In addition, the selected panel of epitope/cytoplasmic markers was qualitatively assessed by immunocytochemistry and photographed using a fluorescence microscope (Nikon, Tokyo, Japan). Briefly, MPCs at a density of 1 × 10^4^ were seeded per well of an 8-well chamber slide and cultured with a growth medium under normal conditions till they attained 70% confluence. At the time, they were washed with DPBS, fixed with 3.7% paraformaldehyde solution, and permeabilized with 0.1% Triton X-100. Following this, they were incubated with 1% BSA and tagged with antihuman primary antibodies such as CD105, CD146, CD166, and CD271. The following day, MPCs were incubated with FITC-conjugated secondary antibody and counterstained with 1 µg/mL of 4′,6-diamidino-2-phenylindole (DAPI). After which, the slides were mounted with a coverslip and observed under the microscope.

### 4.9. Phenotypic Plasticity

MPCs were induced to differentiate into osteocytes, adipocytes, and chondrocytes to determine phenotypic plasticity. Osteogenic, adipogenic, and chondrogenic differentiation was performed in monolayer cultures. Briefly, 2 × 10^4^ cells were seeded per well of a 12-well culture dish and cultured in DMEM/F12 medium supplemented with 10% FBS until they reached 70–80% confluency. Thereby, the media were changed every 3 days with appropriate differentiation media for 28 days. Specific differentiation media were prepared as described previously [21].

### 4.10. Chondrogenic Differentiation of MPCs in 3D-Mass Culture and Pellet Culture

OA and RA MPCs at a high density of 2 × 10^6^ cells were seeded per well of a 16-well chamber slide. The culture was further supplemented with chondrogenic induction media for 28 days. Post culture duration the mass cultures were immunostained with chondrocyte-specific markers (COL2-α1 and ACAN), counterstained with 10 µL/mL of PI, and observed and photographed under the fluorescence microscope.

OA-MPCs and RA-MPCs at 70–80% confluency were trypsinized, and 2 × 10^6^ cells were pelleted by centrifugation at 3000 rpm for 5 min. Cell pellets were grown in 1.8 mL Eppendorf tubes nourished with chondrogenic induction media for 28 days. The pellets were then frozen at −80 °C for downstream gene expression analysis of chondrogenic markers.

### 4.11. Statistical Analysis

All experiments were performed in replicates of three, and the data obtained were analysed using GraphPad Prism 9.12. Results are expressed as mean ± standard deviation. Prior to employing parametric tests, the normality of data distribution was evaluated using the Shapiro–Wilk test. For normally distributed data, comparisons between two groups were made using Student’s *t*-test, while differences among multiple groups were assessed through one-way analysis of variance (ANOVA) followed by Tukey’s post hoc test. In instances where the data did not satisfy the normality assumption, appropriate non-parametric tests were applied. Statistical significance was assessed at *p* < 0.05.

## 5. Conclusions

Although the present study has successfully characterised MPCs from two pathological conditions, such as OA and RA, further studies are warranted in healthy articular cartilage to characterise the properties of native MPCs. Importantly, before the differential diagnosis of OA and RA-MPCs as unaffected, unaltered, and uninvolved in the underlying condition, detailed molecular profiling should be performed using transcriptomic gene analysis.

## Figures and Tables

**Table 1:** List of genes with oligomer sequences used for RT-qPCR.

Genes	Primer Sequences (5′-3′)
*β-Actin*	F: TCCTTCCTGGGCATGGAG R: AGGAGGAGCAATGATCTTGATCTT
*18s RNA*	F: GTAACCCGTTGAACCCCATT R: CCATCCAATCGGTAGTAGCG
*IFNB1*	F: GGTTACCTCCGAAACTGAAGA R: CCTTTCATATGCAGTACATTAGCC
*HBB*	F: GCTTCTGACACAACTGTGTTCACTAGC R: CACCAACTTCATCCACGTTCACC
*SIRT1*	F: TAATAGAGTGGCAAAGGAGCAG R: TACTGCCACAAGAACTAGAGGA
*TP53*	F: TGCTCAAGACTGGCGCTAAA R: CAATCCAGGGAAGCGTGTCA
*CDK1*	F: GCACTTTGATTAGCAGCGGA R: GAAAGACAACTACTCCCAGC
*MKI67*	F: GAGAATCTGTGAATCTGGGTAA R: CAGGCTTGCTGAGGGAAT
*TP16*	F: AACCTCGGGAAACTTAGATC R: TCTACGTTAAAAGGCAGGAC
*CD29*	F: GAAGGGTTGCCCTCCAGA R: GCTTGAGCTTCTCTGCTGTT
*CD44*	F: CAGCTCATACCAGCCATCCA R: GCTTGATGACCTCGTCCCAT
*CD200*	F: TGGAGAGGCTGACTCTGACCA R: GCTGCCATGACCCAAACCAG
*CD271*	F: AGGGAGGAATCGAGGAACCA R: TGAGCTTCGAAAACCCCTCC
*HLA-DR*	F: CATTTCTTCAATGGGACGGAGC R: TGGCTGTTCCAGTACTCCTCA
*NANOG*	F: CCTGTGATTTGTGGGCCTG R: GACAGTCTCCGTGTGAGGCAT
*SOX2*	F: ACACCAATCCCATCCACACT R: GCAAACTTCCTGCAAAGCTC
*SSEA4*	F: TAAGGACATCGCCTACCAGCTC R: TCTTCCAGGTGTCAACGAGGT
*RUNX2*	F: AACTTCCTGTGCTCGGTGCTG R: GGGGAGGATTTGTGAAGACGG
*BGLAP*	F: CGCAGCCACCGAGACACCAT R: GGGCAAGGGCAAGGGGAAGA
*SPARC*	F: GTGCAGAGGAAACCGAAGAG R: AAGTGGCAGGAAGAGTCGAA
*ALP*	F: TAAGGACATCGCCTACCAGCTC R: TCTTCCAGGTGTCAACGAGGT
*ITGA1*	F: AGAGCCTGCGCAATGGAATA R: TTGGGTTGGAAGACTGCTGA
*ITGA2*	F: TCGCCGAACCAAAGTGGATT R: GGGGCAGAGATAGTGGGAGA
*IBSP*	F: ACAACACTGGGCTATGGAGA R: CCTTGTTCGTTTTCATCCAC
*TGFA*	F: GGCTTCTTCAGGACAGCACT R: CCCAAGCAGACGGAGTTCTT
*BMP2*	F: ATGGATTCGTGGTGGAAGTG R: GTGGAGTTCAGATGATCAGC
*BMP6*	F: TGTGACTGGGAAGGCAATTTCA R: CGCCCACACCACGACA
*LPL*	F: AAAGCCCTGCTCGTGCTGAC R: TAAACCGGGCCACATCCTGT
*AP2*	F: TGAGATTTCCTTCATACTGGG R: TGGTTGATTTTCCATCCCATT
*PPAR-γ*	F: CGACCAGCTGAATCCAGAGT R: GATGCGGATGGCCACCTCTT
*ACAN*	F: AGTTCTCAAATTGCATGGGGTGTC R: AGTTCTCAAATTGCATGGGGTGTC
*COL2-α1*	F: CAGCCGCTTCACCTACAGC R: TTTTGTATTCAATCACTGTCGCC
*ACAN*	F: AGTTCTCAAATTGCATGGGGTGTC R: AGTTCTCAAATTGCATGGGGTGTC
*FGFR3*	F: AGGAGCTCTTCAAGCTGCTG R: ACAGGTCCAGGTACTCGTCG
*TGF-β3*	F: GGTTTTCCGCTTCAATGTGT R: TATAGCGCTGTTTGGCAATG
*ANXA6*	F: TTGCTGCTGGGCTAACGG R: GGAAGTCATGGATGGAGCCC
*CNTN1*	F: CTGTCAAGTAGCCAGGGTGG R: CACCTGTTCCCTGCTCCTG
*MATN1*	F: GCGTTCGGCCTGTTGAATTT R: TTCACGGTGCTGGCATAGTT
*TGF-β1*	F: CTGTCCAACATGATCGTGCG R: TGACACAGAGATCCGCAGTC
*VIM*	F: TCCGCACATTCGAGCAAAGA R: TGATTCAAGTCTCAGCGGGC
*SOX9*	F: CCCAACGCCATCTTCAAGG R: CTGCTCAGCTCGCCGATGT

## Data Availability

The datasets generated and/or analyzed during the current study are included in this article. Further inquiries can be directed to the corresponding author.
